# A health promotion behavior intervention for women with polycystic ovary syndrome experiencing infertility: a randomized controlled trial

**DOI:** 10.3389/fpubh.2026.1904708

**Published:** 2026-09-09

**Authors:** Pingpei Dong, Yongjie Tang, Ting Wang, Yunxi Xue, Xunxun Ying, Yang Jin

**Affiliations:** Department of Gynecology and Obstetrics, Hangzhou Hospital of Traditional Chinese Medicine, Affiliated Hangzhou Traditional Chinese Medicine (TCM) Hospital of Zhejiang Chinese Medical University, Hangzhou, China

**Keywords:** behavior change wheel, health promotion behavior, infertility, polycystic ovary syndrome, self-efficacy

## Abstract

**Objective:**

This study aimed to evaluate the effectiveness of an intervention based on the behavior change wheel (BCW) in improving health promotion behaviors, self-efficacy, and pregnancy rates among women with polycystic ovary syndrome (PCOS) experiencing infertility.

**Methods:**

This randomized controlled trial included 66 PCOS patients with infertility recruited from the gynecology outpatient department of a tertiary hospital in Hangzhou, China. Participants were randomly assigned to either the experimental group (*n* = 33), which received a 12-week health promotion behavior intervention based on the BCW, or the control group (*n* = 33), which received usual care. Outcomes were measured at baseline and immediately after the intervention.

**Results:**

A total of 62 participants were included in the full analysis set, and 56 in the per-protocol set. Health promotion behaviors improved significantly from pre-intervention to post-intervention in both groups (*p* < 0.05). At post-intervention, the experimental group showed significantly greater improvement compared to the control group (*p* < 0.05). Self-efficacy was also significantly higher in the experimental group than in the control group at post-intervention (*p* < 0.05). However, the difference in pregnancy rate between the two groups was not statistically significant (*p* > 0.05).

**Conclusion:**

The BCW-based intervention program showed promise in improving health promotion behaviors and self-efficacy among PCOS patients with infertility over 12 weeks, although its effect on pregnancy rate was not statistically significant. Longer-term studies are warranted to evaluate sustained benefits and reproductive outcomes.

**Clinical trial registration:**

https://www.chictr.org.cn, identifier (ChiCTR2500103129).

## 1 Introduction

Polycystic ovary syndrome (PCOS) is the most common endocrine disorder affecting women of reproductive age, with a lifespan impact from adolescence to menopause and a prevalence of 10–13% (1, 2). Affected women present a range of clinical manifestations, including hyperandrogenism, oligo-ovulation or anovulation, and polycystic ovaries (3). Furthermore, patients with PCOS have higher rates of obesity (4), type 2 diabetes mellitus (5), and anxiety and depression (6) compared to healthy populations. Infertility, defined as the failure to achieve pregnancy after at least 12 months of regular, unprotected sexual intercourse (7), affects approximately 13% of the global population (8). PCOS is the most common cause of anovulatory infertility (9).

Women with PCOS experiencing infertility face a distinct set of psychological and treatment-related challenges that distinguish them from PCOS patients without fertility difficulties. Psychologically, these women experience fertility-related stress at a moderate-to-high level (10), along with diminished quality of life, heightened anxiety and depressive symptoms, and psychosexual disturbances (11–13). In addition to this psychological burden, the treatment pathway for this subgroup poses particular difficulties. The international and Chinese guidelines recommend lifestyle intervention as the first-line foundational management for PCOS patients experiencing infertility, encompassing weight management, dietary control, regular exercise, and other multifaceted strategies (11, 14). Achieving meaningful clinical improvement requires multidisciplinary collaboration to develop and implement systematic care plans that optimize body composition, reduce body weight, maintain a positive lifestyle and emotional state, and ultimately restore ovulation—thereby improving both reproductive outcomes and metabolic profiles (15).

Health promotion behavior (HPB) refers to the positive, multidimensional actions individuals take to improve and enhance their health status (16). The latest evidence-based management guidelines for PCOS recommend healthy lifestyles for all patients and emphasize the need for lifestyle adjustments and adherence to HPBs for those experiencing infertility during pregnancy preparation (11). The recommended HPBs for PCOS patients encompass five dimensions: health responsibility, dietary nutrition, exercise, interpersonal support, and emotional management (17).

A cross-sectional survey by Liu et al. (18) on the level of HPBs among PCOS patients revealed that 40.6% had poor HPBs, 31.8% were at a moderate level, and only 20.3 and 7.3% achieved good and excellent levels, respectively. Other studies have also indicated that overall HPBs in PCOS patients are only at a moderate level (19, 20). Therefore, the level of HPBs among PCOS patients with infertility urgently needs improvement.

**Figure 1:**
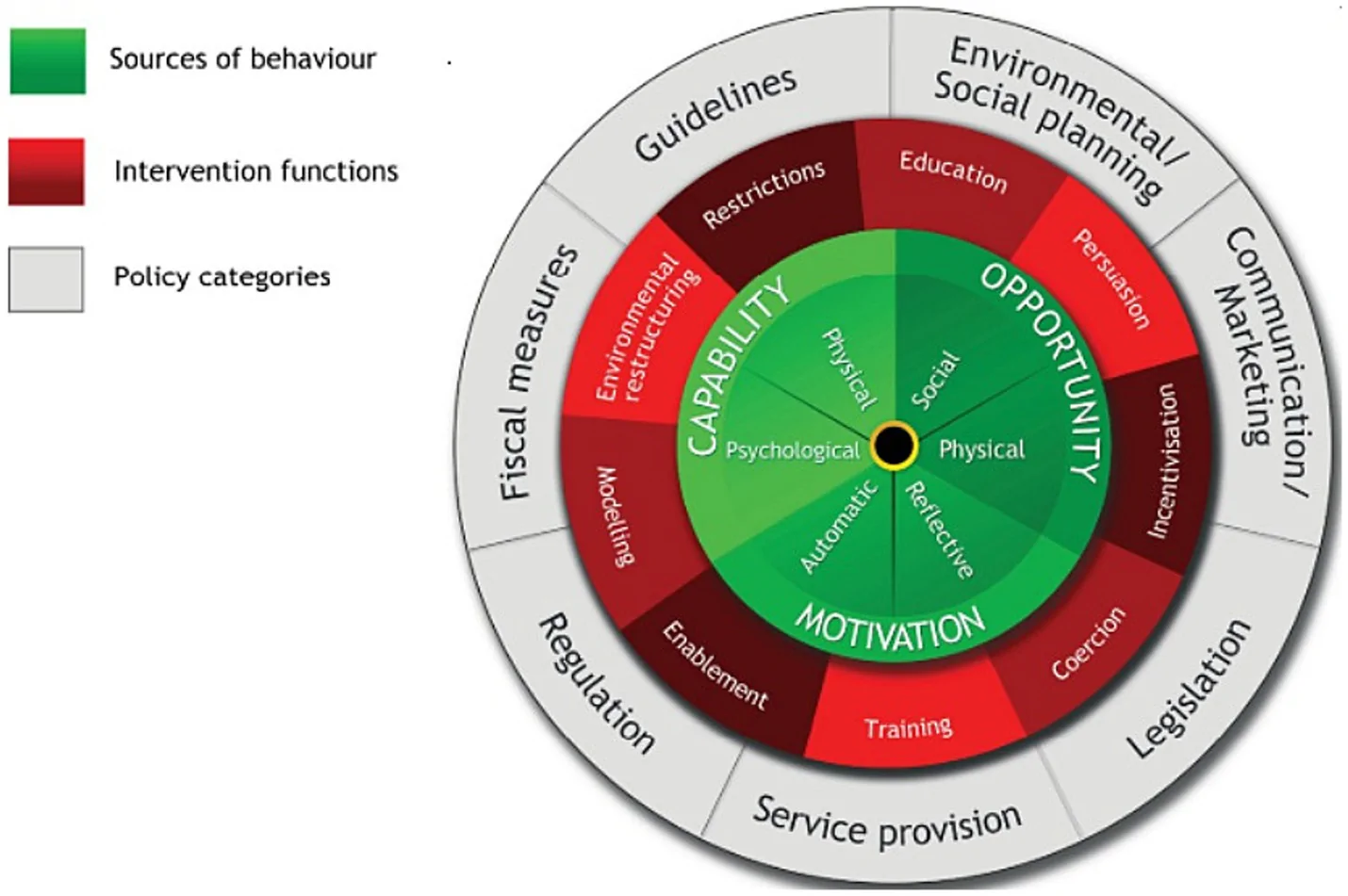
The Behavior change wheel. Reprinted from Michie et al. (21). Licensed under CC BY 2.0.

The Behavior Change Wheel (BCW) theory, proposed by Michie et al. (21) in 2011, assists researchers in analyzing the mechanisms of behavior and their influencing factors, providing guidance for the design and implementation of intervention programs. It is currently one of the most comprehensive health behavior theories for explaining behavior. The theory is structured as a three-layer hub-and-spoke model. From the center outward, the core layer is the Capability, Opportunity, Motivation– Behavior (COM-B) model, which explains the formation of the target behavior. The middle layer encompasses nine intervention functions: education, persuasion, incentivization, coercion, training, restriction, environmental restructuring, modeling, and enablement. The outer layer comprises seven policy categories: communication/marketing, guidelines, fiscal measures, regulation, legislation, environmental/social planning, and service provision. The specific structure is shown in Figure 1.

The BCW theory has been successfully applied in promoting breastfeeding (22) and facilitating health behaviors in post-PCI patients with coronary heart disease (23). Several theory-driven behavioral interventions have been previously conducted among women with PCOS. Hajivandi et al. (24) implemented a four-session educational program based on the Theory of Planned Behavior—including lectures, group discussions, and role-playing—among 72 Iranian adolescents and young adults with PCOS, and found significant improvements in nutritional behaviors that contributed to better nutritional and reproductive health. Du et al. (25) applied an intervention grounded in the Health Action Process Approach (HAPA) to promote dietary control behaviors in obese women with PCOS, reporting improvements in dietary adherence, serum free testosterone levels, and 2-h post-load glucose levels. Similarly, Miao (26) used the Health Belief Model to guide an intervention for overweight or obese women with PCOS experiencing infertility, demonstrating reductions in body weight and body mass index, along with enhanced health beliefs and lifestyle improvements. While these studies confirm the value of theory-driven approaches over routine education, their theoretical frameworks—the Theory of Planned Behavior, the Health Belief Model, and the HAPA—predominantly focus on modifying individual cognitive and motivational factors, with limited systematic attention to external environmental constraints and the practical capabilities required for sustained behavior change. These limitations suggest the need for a more comprehensive theoretical framework that simultaneously addresses capability, opportunity, and motivation—precisely the integrative structure offered by the BCW theory—to develop a systematic and multidimensional health promotion behavior intervention specifically tailored for women with PCOS experiencing infertility. However, the BCW theory has not yet been applied to HPB interventions for women with PCOS experiencing infertility.

Within the COM-B model, self-efficacy—an individual’s confidence in their ability to perform specific actions—is a core element of reflective motivation, as it involves self-beliefs and perceived behavioral control that shape intentional decisions to act. Low self-efficacy can undermine the translation of knowledge and motivation into sustained behavior change. Several BCW intervention functions directly target self-efficacy: persuasion and incentivization reinforce positive beliefs and provide encouragement; and modeling offers peer examples to learn from. Enhanced self-efficacy, in turn, supports both the initiation and maintenance of health promotion behaviors. Thus, self-efficacy is included as a study outcome because it represents a theoretically relevant factor that may mediate the pathway from the BCW-based intervention to improved HPBs.

Therefore, this study aimed to explore the effectiveness of an intervention based on the BCW theory in improving HPBs, self-efficacy, and pregnancy rates in this population. Specifically, we hypothesized that, compared with the control group receiving usual care, the experimental group receiving the BCW-based intervention would show (1) significantly greater improvements in HPBs, (2) significantly greater increases in self-efficacy, and (3) a higher pregnancy rate after the 12-week intervention period.

## 2 Methods

### 2.1 Study design

This was a single-center, randomized, parallel-group, controlled trial conducted from June 2025 to December 2025 at a tertiary hospital in Hangzhou, China. The trial used blinded outcome assessment (assessor-blinded design), with data collectors and analysts masked to group allocation, while participants and intervention providers were not blinded due to the behavioral nature of the intervention.

### 2.2 Sample

The sample size was calculated using the formula for comparing two sample means, with the total score of the Health Promotion Lifestyle Profile for PCOS Patients as the primary outcome indicator. The formula was as follows:


$$n_{1}=n_{2}=2{\left[(\mu_{\alpha }+\mu_{\beta })/(\delta /\sigma )\right]}^{2}$$

With *α* = 0.05, *β* = 0.10, *μ*_α_ = 1.96, *μ*_β_ = 1.282, using a two-sided test. Based on previous literature (17), the standard deviation of the health promotion lifestyle score for PCOS patients was 14.273. The anticipated mean difference between the two groups post-intervention was set at 13 points, which was determined based on clinical experience and expert consensus among our research team (including senior nurses and gynecologists specializing in PCOS management). This 13-point difference was considered clinically meaningful because it would move a patient from the moderate range (87–104) to the high-level range (105–165) on the PCOS-HPLP scale, representing a practically significant improvement in health promotion behaviors that would be perceivable in daily life. Considering a 20% attrition rate and clinical feasibility, the final total sample size was determined to be 66 cases, with 33 cases in each group (intervention and control).

**Figure 2:**
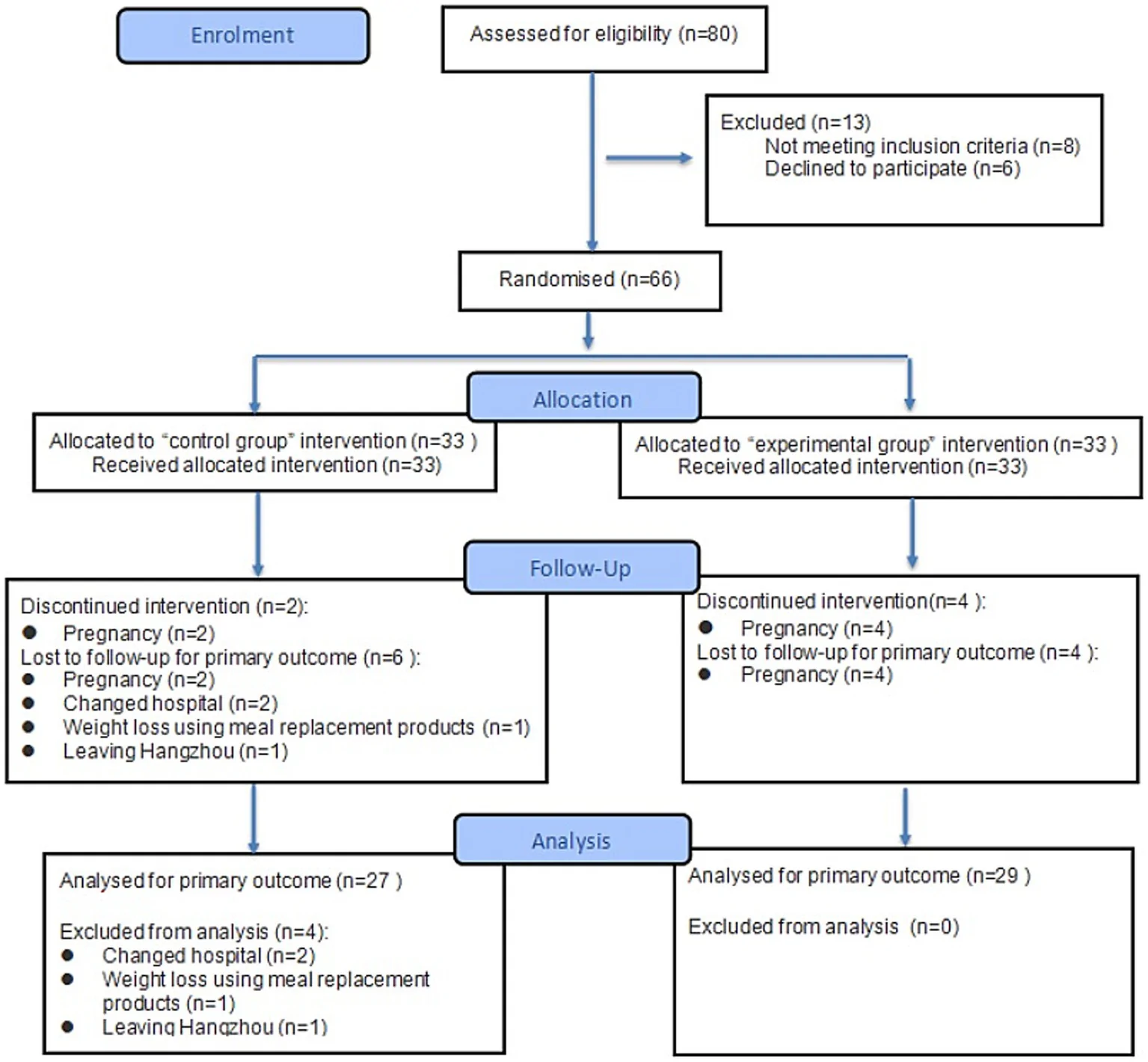
Flowchart of the study, adapted from the CONSORT 2025 Statement (https://www.consort-spirit.org/).

Patients were required to meet the following criteria: (1) meet the Rotterdam (2003 revised) diagnostic criteria for PCOS (27); (2) meet the diagnostic criteria for infertility according to the 2019 guidelines (28); (3) be at least 20 years old; (4) have a total score of ≤104 on the Health Promotion Lifestyle Profile for PCOS Patients; (5) have at least a primary school education level and normal communication skills. Patients were excluded if they had (1) severe cognitive impairment or neuropsychiatric disorders; (2) other severe chronic diseases affecting quality of life (e.g., malignancy); (3) infertility due to male factors, tubal factors, cervical factors, or other causes; (4) ovulation disorders caused by hyperprolactinemia, premature ovarian failure, etc.; (5) recently experienced serious life events such as major illness or death of a first-degree relative, or significant financial loss; (6) were participating in other similar studies. Participants could withdraw if they (1) chose to do so voluntarily; (2) were lost to follow-up; (3) could not be contacted. The study could be terminated for a patient if they (1) developed a serious physical illness during the intervention; (2) became pregnant during the intervention. Participant inclusion and randomization are represented in Figure 2.

### 2.3 Randomization and blinding

An independent researcher not involved in the study used SPSS 26.0 to randomly generate a sequence of numbers from 01 to 66. Odd numbers were assigned to the experimental group, and even numbers to the control group. Eligible patients were randomly assigned to the two groups in a 1:1 ratio. Opaque envelopes were used to conceal the random sequence. Participant allocation was performed by research personnel not involved in recruitment or intervention. Due to the behavioral nature of the intervention, blinding of participants and intervention providers was not feasible. Participants in the experimental group inevitably knew they were receiving additional counseling and support beyond routine care, and the nurses delivering the health education were aware of the participants’ group assignments. However, outcome assessors (data collectors) and data analysts were successfully blinded to group allocation throughout the study. Data collectors, who received uniform training, were prohibited from asking patients about their group assignment or specific interventions. Additionally, coded anonymous data were used to ensure blinding during data analysis. Therefore, this trial is best characterized as an assessor-blinded (outcome assessor-blinded) randomized controlled trial.

### 2.4 Intervention

#### 2.4.1 Control group

The control group received routine health education delivered by outpatient nurses following standard protocols. The content included basic knowledge of PCOS and guidance on PCOS health promotion behaviors (diet, exercise, emotional management). The specific health education content for the control group is detailed in Table 1.

**Table 1:** Routine health education content for the control group.

Topic	Content
Basic PCOS Knowledge	1. Etiology: The cause of PCOS is currently unclear, possibly related to genetic and environmental factors. 2. Clinical manifestations: PCOS often begins in adolescence, with main symptoms including menstrual disorders, infertility, hirsutism, acne, obesity, and acanthosis nigricans. 3. Diagnostic criteria: If a patient has oligomenorrhea, amenorrhea, or irregular uterine bleeding, this is a necessary prerequisite. A definite diagnosis of PCOS can be made if, on this basis, they also meet one of the following two criteria: (1) clinical or laboratory signs of hyperandrogenism; (2) polycystic ovarian morphology on ultrasound. Other causes of hyperandrogenism or ovulatory dysfunction must be excluded before diagnosis. 4. Treatment approaches: Currently recommended treatment strategies for PCOS primarily include: (1) Lifestyle intervention as the first-line fundamental management; (2) Medication to help restore regular menstrual cycles, control androgen levels, and improve insulin sensitivity; (3) For patients with fertility desires, ovulation induction may be initiated after completing lifestyle interventions, controlling androgen levels, and improving insulin resistance.
Diet Management	1. Limit intake of high-calorie, high-fat, high-cholesterol, and high-salt foods (e.g., fatty meat, barbecue, spicy hot pot, fried foods). 2. Control consumption of sugar and sugary products (e.g., desserts, sugary drinks, highly processed high-sugar fast foods). 3. Appropriately increase the proportion of whole grains and legumes (e.g., corn, quinoa) in staple foods. 4. Ensure adequate daily intake of fresh vegetables (approximately 500 g). 5. Choose high-quality protein sources daily, prioritizing eggs, low-fat dairy, fish and shrimp, lean meat, and soy products.
Exercise Management	1. Aim for aerobic exercise at least 5 days per week, at least 30 min per session (e.g., brisk walking, running, cycling, swimming). 2. Schedule resistance training about 3 times per week, on non-consecutive days, for 10–20 min per session (e.g., sit-ups, push-ups). 3. Perform a warm-up for about 3–5 min before exercise (e.g., mobilizing wrists, ankles, knees). 4. Perform cool-down stretches for 5–10 min after exercise, focusing on major muscle groups (e.g., front/back of thighs, ankles). 5. Monitor physical responses during exercise; stop immediately if symptoms like dizziness, blurred vision, or palpitations occur. 6. Avoid prolonged sitting; get up and move for 5–10 min every 1–2 h; aim for approximately 10,000 steps per day.
Emotional Management	1. Manage negative emotions related to disease symptoms (e.g., hirsutism, acne, alopecia, infertility) through positive self-regulation strategies (e.g., confiding in relatives/friends, distraction). 2. If experiencing significant anxiety, depression, or sleep disturbances (e.g., difficulty falling asleep, persistent fatigue upon waking) that are difficult to manage alone, seek timely professional medical help.

#### 2.4.2 Experimental group

In addition to the routine care received by the control group, the experimental group received an intervention based on the BCW theory. The specific implementation process is detailed below (Table 2).

**Table 2:** BCW-based intervention for the experimental group.

Intervention level	Intervention function	Specific intervention measures	Time (12 weeks)
1. Capability	1.1 Education	1.1.1 Provide patients with the “PCOS Patient Health Education Manual,” covering a brief introduction to PCOS, including etiology, symptoms, common tests, treatments, and pregnancy preparation methods.	Week 1
		1.1.2 Explain PCOS definition, etiology, symptoms, health hazards, diagnostic methods, and treatment measures in detail to patients and their families via Tencent Meeting.	Week 1
		1.1.3 Use easy-to-understand videos or animations with subtitles to explain the menstrual cycle, ovulation process, and how PCOS affects ovulation leading to infertility.	Week 1
	1.2 Training	1.2.1 Explain the energy-restricted balanced diet combined with low glycemic index (GI) dietary pattern via Tencent Meeting; teach use of the food exchange list and selection of low-GI foods based on the food GI value table.	Week 2
		1.2.2 Guide patients in using “Boohee Health” or other calorie management apps to check food calories and components, calculate and record daily intake.	Week 2
		1.2.3 Guide patients on warm-up exercises before formal exercise and stretching afterward. Explain exercise targets: Non-overweight/obese patients: at least 150–300 min of moderate-intensity or 75–150 min of vigorous-intensity aerobic activity per week, plus muscle-strengthening activities on 2 non-consecutive days per week (resistance/flexibility, 10–20 min each). Overweight/obese patients: at least 250 min of moderate-intensity or 150 min of vigorous-intensity aerobic activity per week, plus muscle-strengthening activities on 2 non-consecutive days per week (resistance/flexibility, 10–20 min each).	Week 2
		1.2.4 Teach patients relaxation training, Five Elements Music Therapy, and the traditional Chinese Qigong “Liu Zi Jue” for emotional management.	Week 3
2. Motivation	2.1 Education	2.1.1 Develop a one-week meal plan: daily diet composition of carbohydrates 55–65%, fat 20–25%, protein 10–15% of total energy, prioritizing low-GI carbohydrates.	Week 3
		2.1.2 Provide syndrome-specific dietary adjustments based on TCM pattern differentiation under the guidance of a chief TCM physician.	Week 3
		2.1.3 Create a one-week exercise plan for each patient based on the exercise targets taught in Week 2.	Week 3
		2.1.4 Guide patients to progress gradually based on their baseline fitness and tolerance, increasing exercise volume by 5% per week in 10-min increments until reaching or exceeding the target.	Week 2
		2.1.5 Inform patients of target heart rate during exercise: Moderate intensity: (220 – age) × (55–70%); Vigorous intensity: (220 – age) × (70–90%); For those with chronic diseases like diabetes/hypertension: target heart rate = (170 – age).	Week 2
		2.1.6 Advise patients to use fitness trackers (e.g., activity wristbands) to monitor daily activity volume and intensity, and guide them to keep an exercise diary.	Week 2
		2.1.7 Explain exercise precautions: prioritize safety, warm up adequately, progress gradually, avoid overexertion; wear appropriate footwear and clothing to prevent injury; exercise 0.5–1.0 h after meals; stop immediately and seek researcher assistance if abnormal symptoms occur (e.g., dizziness, palpitations, cold sweat, dyspnea, sudden numbness or pain in limbs).	Week 2
	2.2 Enablement	2.2.1 Ask patients to identify their current HPB issues and the gap between their current state and target behaviors.	Week 3
		2.2.2 Ask patients to list potential obstacles to performing target behaviors and assist them in developing specific countermeasures.	Week 3
		2.2.3 Send patients electronic diet and exercise log sheets, requesting them to record diet and exercise for at least 4 days per week (including at least one weekend day). Researchers analyze goal completion weekly and provide feedback.	Weeks 4–12
	2.3 Persuasion	2.3.1 Inform patients of the importance of HPBs for pregnancy preparation and long-term health in PCOS, illustrated with real-life examples.	Week 1
		2.3.2 Use motivational interviewing techniques to affirm patients’ self-ability and dispel self-doubt.	Weeks 4–12
	2.4 Incentivization	2.4.1 Ask the patient’s primary caregiver to participate in the behavior change plan as a supervisor and supporter.	Week 1
		2.4.2 Ask the primary caregiver to provide verbal praise and material rewards (ensuring rewards do not conflict with health goals) upon completion of weekly behavioral goals.	Week 1
		2.4.3 Guide patients to discover their strengths and use them for “self-affirmation” to build confidence.	Weeks 4–12
	2.5 Modeling	2.5.1 Help patients form behavior change support groups with peers, where members share their recent HPB experiences for mutual support and supervision.	Week 1
3. Opportunity	3.1 Environmental Restructuring	3.1.1 Based on weekly goal completion, follow-up findings, and patient needs, push relevant content via WeChat at fixed times each week.	Weeks 4–12
		3.1.2 Conduct a fixed weekly evening (19:00–20:00) healthcare team-patient exchange in a WeChat group to understand patient behavior changes, assess comprehension of pushed content, and answer patient questions.	Weeks 4–12
		3.1.3 Guide patients to post daily diet, exercise, and emotional management goals in visible daily places (e.g., at home, workstation) or set them as phone wallpapers.	Week 3
	3.2 Modeling	3.2.1 Acknowledge patients with high goal completion during the weekly evening WeChat exchange, inviting them to share their behavior change experiences and methods with others, providing role models and positive examples to enhance patient confidence.	Weeks 4–12
	3.3 Enablement	3.3.1 Use telephone, WeChat, or face-to-face follow-up to inquire about patients’ HPB practices over the past 2 weeks and problems encountered, answering questions and addressing confusion.	Weeks 4–12
		3.3.2 Assess patients’ recent psychological status during follow-up. If patients experience frustration due to failure to meet behavioral goals, use motivational interviewing techniques to provide timely psychological support and encouragement to restore hope and confidence. If emotional problems are severe, recommend evaluation at a mental health department.	Weeks 4–12

### 2.5 Data source

#### 2.5.1 Basic characteristics

A general information questionnaire designed by the research team was used, covering sociodemographic data (age, education, marital status, residence, total monthly household income, payment method for medical expenses) and disease-related data (disease duration, obstetric history, height, weight, waist circumference, hip circumference).

#### 2.5.2 Health promotion lifestyle profile for polycystic ovary syndrome patients

Developed by Zhang et al. (17) based on Pender’s Health Promotion Model. It consists of 33 items across five dimensions: interpersonal support, health responsibility, emotional management, dietary nutrition, and exercise. It uses a Likert 5-point scale (1 = never, 2 = rarely, 3 = sometimes, 4 = often, 5 = always), all positively scored. Higher scores indicate better HPB levels. Scores 33–86 indicate low level, 87–104 moderate level, 105–165 high level. The Cronbach’s α coefficient for the total scale is 0.930, and for subscales ranges from 0.795 to 0.891, as reported in the original validation study (12). In our current sample (baseline data, *n* = 62, full analysis set), the Cronbach’s α coefficients were 0.764 for the total scale and 0.682 (dietary nutrition), 0.765 (exercise), 0.461 (health responsibility), 0.251 (interpersonal support), and 0.749 (emotional management) for the respective dimensions. The lower values observed for the health responsibility and interpersonal support dimensions are likely attributable to sample-specific variability, as Cronbach’s α is known to be sample-dependent and sensitive to sample size and the number of items per dimension (29, 30). The original validation values, based on a larger sample (*n* = 316), are provided as the primary reference for the scale’s established psychometric properties.

#### 2.5.3 General self-efficacy scale

Originally developed by Professor Ralf Schwarzer and colleagues at the Free University of Berlin in 1981. The Chinese version (GSES) was first used by Zhang Jianxin and Schwarzer (40) among Hong Kong first-year university students. It contains 10 items assessing individual confidence in facing setbacks or difficulties, using a Likert 4-point scale (1 = not at all true, 2 = hardly true, 3 = moderately true, 4 = exactly true). The Chinese version has demonstrated good reliability and validity (31), with a Cronbach’s α coefficient of 0.96.

#### 2.5.4 Pregnancy rate

Pregnancy was confirmed by serum β-human chorionic gonadotropin (β-hCG) testing, with a level of ≥10 IU/L defined as a positive pregnancy diagnosis. The pregnancy rate was calculated as the ratio of the number of confirmed pregnancies during the intervention period to the number of cases in the Full Analysis Set (FAS).

### 2.6 Data collection

One-on-one data collection was conducted before and after the intervention. The principal investigator recruited and trained a research assistant with a master’s degree in nursing and nursing research experience. This assistant remained blinded to participant group allocation. Before baseline data collection (T0), the study’s purpose and procedures were explained in detail, emphasizing anonymous data processing and strict confidentiality. Informed consent was obtained after ensuring participant understanding and trust. All questionnaires were completed in a quiet, comfortable environment. Questionnaires were collected and checked for completeness on-site; omissions were addressed by re-emphasizing the importance of complete data and confidentiality. Data were collected at baseline (T0) and immediately post-intervention (T1), with each session lasting 5–10 min, ensuring timeliness and completeness.

### 2.7 Data analysis

#### 2.7.1 Analysis datasets

##### 2.7.1.1 Full analysis set (FAS)

All patients randomized who received all or part of the assigned intervention and had evaluable intervention effects (e.g., PCOS-HPLP score, GSES score, pregnancy rate), following the Intention-To-Treat (ITT) principle.

##### 2.7.1.2 Per protocol set (PPS)

Randomized patients meeting inclusion/exclusion criteria, with complete data for each evaluation index, who received all assigned intervention measures.

#### 2.7.2 Statistical analysis methods

Data entry used Excel; statistical analysis used SPSS 26.0.

(1) Descriptive statistics: Mean and SD for normally distributed quantitative data; median and IQR for non-normally distributed quantitative data; frequency and percentage for categorical data.

(2) Comparisons between groups: Independent samples t-test, paired t-test, independent samples rank sum test, paired rank sum test, and chi-square test.

(3) Significance level α = 0.05.

### 2.8 Ethical considerations

This study was conducted following the Helsinki Declaration and received approval from the Ethics Committee of the Hangzhou TCM Hospital Affiliated to Zhejiang Chinese Medical University (Approval No. 2024KLL166). The study was registered with the Chinese Clinical Trial Registry (ChiCTR2500103129; https://www.chictr.org.cn). The study strictly adhered to ethical principles, and informed consent was obtained from all participants.

## 3 Results

### 3.1 Sample distribution

After rigorous screening, 66 patients (33 per group) were included. By study end, 4 patients in the control group were excluded (2 were lost to follow-up at our hospital, 1 used meal replacements for weight loss after enrollment, 1 moved away for work), resulting in a total attrition rate of 6%. These were not included in the FAS or statistical analysis. During the study, 4 patients in the experimental group and 2 in the control group discontinued due to pregnancy and were not included in the PPS. Specific distribution is shown in Table 3.

**Table 3:** Sample distribution (cases).

Group	*N*	Full analysis set (FAS)	Per protocol set (PPS)
Experimental	33	33	29
Control	33	29	27

In the control group, participants 08 and 26 were lost to follow-up, participant 15 used meal replacements for weight loss, participant 44 moved away for work; these 4 were not included in FAS or statistics. In the experimental group, participants 07, 12, 18, and 21 discontinued due to pregnancy; in the control group, participants 06 and 22 discontinued due to pregnancy; these 6 were not included in PPS.

### 3.2 Demographic and baseline characteristics

Chi-square tests compared categorical variables (ethnicity, residence, occupation, monthly household income, payment method, education, religion, history of pregnancy, history of delivery, current infertility treatment, BMI achieved-target status, waist-to-hip ratio achieved-target status). Rank sum tests compared marriage duration, time since PCOS diagnosis, time since infertility diagnosis, time trying to conceive, and pre-intervention HPB score. Independent samples *t*-test compared age and pre-intervention self-efficacy score. Results showed no statistically significant differences in demographic or baseline characteristics between groups (*p* > 0.05) (see Table 4).

**Table 4:** Comparison of demographic and baseline characteristics between groups [*n*(%)] (FAS).

Variable	Category	Experimental (*n* = 33)	Control (*n* = 29)	*t/χ* ^2^ */Z*	*P*
Age (years)		30.85 ± 3.26	31.03 ± 4.56	−0.186^a^	0.853
Ethnicity	Han	33 (100)	28 (96.6)	–^b^	0.468
	Other	0 (0.0)	1 (3.4)		
Residence	Rural	7 (21.2)	8 (27.6)	4.865^b^	0.088
	Town	8 (24.2)	13 (44.8)		
	Urban	18 (54.5)	8 (27.6)		
Occupation	Office Employee	18 (54.5)	16 (55.2)	1.944^b^	0.885
	Teacher	5 (15.2)	4 (13.8)		
	Healthcare Worker	0 (0.0)	1 (3.4)		
	Freelancer	7 (21.2)	7 (24.1)		
	Unemployed	3 (9.1)	1 (3.4)		
Monthly Household Income (CNY)	<5,000	1 (3.0)	6 (20.7)	7.393^b^	0.059
	5,001 ~ 10,000	12 (36.4)	14 (48.3)		
	10,001 ~ 20,000	13 (39.4)	6 (20.7)		
	>20,000	7 (21.2)	3 (10.3)		
Medical Payment Method	Self-pay	7 (21.2)	2 (6.9)	3.427^b^	0.112
	Medical Insurance	25 (75.8)	27 (93.1)		
	Publicly Funded	1 (3.0)	0 (0.0)		
Education	Middle School	1 (3.0)	3 (10.3)	3.025^b^	0.417
	High School/Vocational	2 (6.1)	4 (13.8)		
	Bachelor/Associate Degree	27 (81.8)	21 (72.4)		
	Master’s Degree or Higher	3 (9.1)	1 (3.4)		
Religious Beliefs	Yes	2 (6.1)	0 (0.0)	–^b^	0.494
	No	31 (93.9)	29 (100.0)		
Marriage Duration (years)		3.00 (2.00, 4.50)	2.00 (2.00, 5.00)	−0.208^c^	0.835
Time since PCOS Diagnosis (years)		5.00 (2.00, 6.50)	3.00 (1.00, 6.50)	−1.371^c^	0.170
Time since Infertility Diagnosis (years)		2.00 (1.00, 3.00)	1.00 (1.00, 2.50)	−1.290^c^	0.197
Time Trying to Conceive (years,)		3.00 (2.00, 4.00)	2.00 (2.00, 3.50)	−1.651^c^	0.099
Previous Pregnancy	Yes	13 (39.4)	12 (41.4)	0.025^b^	0.874
	No	20 (60.6)	17 (58.6)		
Previous Delivery	Yes	6 (18.2)	5 (17.2)	0.009^b^	0.923
	No	27 (81.8)	24 (82.8)		
BMI (Achieved Target)	Yes	15 (45.5)	14 (48.3)	0.049^b^	0.824
	No	18 (54.5)	15 (51.7)		
Waist-to-Hip Ratio (Achieved Target)	Yes	23 (69.7)	14 (48.3)	2.943^b^	0.086
	No	10 (30.3)	15 (51.7)		
Current Infertility Treatment	Natural Conception	29 (87.9)	25 (86.2)	<0.001^b^	1.000
	Assisted Reproductive Tech.	4 (12.1)	4 (13.8)		
PCOS-HPLP Score		97.00 (90.00, 101.50)	99.00 (92.50, 102.00)	−0.764^c^	0.445
GSES Score		25.82 ± 5.20	25.55 ± 5.79	0.191^a^	0.849

^a^Independent samples *t*-test, ^b^chi-square test, ^c^rank sum test.

### 3.3 Effects of the intervention on health promotion behaviors

Paired t-tests or paired rank sum tests (based on normality of change scores) showed statistically significant changes within both groups from pre- to post-intervention for all dimension scores and total score (all *p* < 0.05). In the experimental group, the mean total score increased from 96.00 (IQR: 90.00–101.50) to 121.00 (IQR: 109.00–129.00), representing a median improvement of 25 points. The control group showed a more modest improvement, from 99.00 (IQR: 94.00–102.00) to 110.00 (IQR: 105.00–113.00), a median increase of 11 points.

Between-group comparisons used independent samples *t*-tests or rank sum tests based on normality. No significant differences were observed between groups at pre-intervention for any score (all *p* > 0.05). At post-intervention, the experimental group demonstrated significantly greater improvements than the control group in the total score (*Z* = −3.119, *p* = 0.002) and in the dietary nutrition (*t* = 2.084, *p* = 0.042), exercise (*t* = 3.010, *p* = 0.005), and health responsibility (*t* = 3.372, *p* = 0.001) dimensions. No significant between-group differences were observed for interpersonal support (*t* = 1.778, *p* = 0.081) or emotional management (*t* = 0.982, *p* = 0.330) (see Table 5 for complete data).

**Table 5:** Comparison of PCOS-HPLP dimension and total scores between groups (mean ± SD)/[*M* (P25, P75)] (PPS).

Dimension	Group	*n*	Pre-intervention	Post-intervention	*t/Z*	*P*
Dietary Nutrition	Experimental	29	19.45 ± 3.69	25.59 ± 4.08	−9.636^c^	<0.001
	Control	27	19.48 ± 3.30	23.41 ± 3.72	−4.299^d^	<0.001
	*t*		−0.035^a^	2.084^a^		
	*P*		0.972	0.042		
Exercise	Experimental	29	21.38 ± 5.36	31.00 ± 7.31	−8.005^c^	<0.001
	Control	27	24.00 ± 4.42	26.52 ± 3.17	−4.782^c^	<0.001
	*t*		−1.987^a^	3.010^a^		
	*P*		0.052	0.005		
Health Responsibility	Experimental	29	22.55 ± 3.21	27.07 ± 3.39	−6.888^c^	<0.001
	Control	27	22.63 ± 2.88	24.48 ± 2.17	−3.312^c^	0.003
	*t*		−0.095^a^	3.372^a^		
	*P*		0.924	0.001		
Interpersonal Support	Experimental	29	13.90 ± 2.06	15.72 ± 2.63	−4.338^c^	<0.001
	Control	27	13.44 ± 1.83	14.63 ± 1.88	−5.380^c^	<0.001
	*t*		0.867^a^	1.778^a^		
	*P*		0.390	0.081		
Emotional Management	Experimental	29	15.86 ± 3.48	19.76 ± 3.78	−6.699^c^	<0.001
	Control	27	16.48 ± 3.63	18.81 ± 3.39	−4.350^c^	<0.001
	*t*		−0.652^a^	0.982^a^		
	*P*		0.517	0.330		
Total Score	Experimental	29	96.00 (90.00, 101.50)	121.00 (109.00, 129.00)	−10.591^c^	<0.001
	Control	27	99.00 (94.00, 102.00)	110.00 (105.00, 113.00)	−9.320^c^	<0.001
	*Z*		−1.224^b^	−3.119^b^		
	*P*		0.221	0.002		

^a^Independent samples *t*-test, ^b^rank sum test,^c^ paired *t*-test, ^d^ paired rank sum test.

### 3.4 Effects of the intervention on self-efficacy

Paired rank sum tests (based on normality of change scores) showed a statistically significant increase in self-efficacy scores in the experimental group, from 26.14 ± 5.05 at baseline to 29.79 ± 4.70 at post-intervention (*Z* = −4.248, *p* < 0.001), representing a mean improvement of 3.65 points. In contrast, the control group showed only a minimal increase from 25.48 ± 5.87 to 26.67 ± 4.32, which did not reach statistical significance (*Z* = −1.758, *p* = 0.079).

Between-group comparisons using independent samples t-tests showed no significant difference at pre-intervention (*t* = 0.450, *p* = 0.655). At post-intervention, the experimental group scored significantly higher than the control group (*t* = 2.587, *p* = 0.012) (see Table 6).

**Table 6:** Comparison of GSES scores between groups (mean ± SD) (PPS).

Variable	Group	*n*	Pre-intervention	Post-intervention	*Z*	*P*
Self-Efficacy	Experimental	29	26.14 ± 5.05	29.79 ± 4.70	−4.248^b^	<0.001
	Control	27	25.48 ± 5.87	26.67 ± 4.32	−1.758^b^	0.079
	*t*		0.450^a^	2.587^a^		
	*P*		0.655	0.012		

^a^Independent samples *t*-test, ^b^paired rank sum test.

### 3.5 Effects of the intervention on pregnancy rate

During the 12-week intervention period, 4 pregnancies (12.1%) were confirmed in the experimental group and 2 pregnancies (6.9%) in the control group, representing an absolute difference of 5.2 percentage points. However, chi-square test showed that this difference was not statistically significant (*χ*^2^ = 0.070, *p* = 0.792) (see Table 7).

**Table 7:** Comparison of pregnancy rates between groups [*n*(%)] (FAS).

Variable	Group	*n*	Pregnant	Not pregnant	*χ^2^*	*P*
Pregnancy Rate	Experimental	33	4(12.1)	29(87.9)	0.070	0.792
	Control	29	2(6.9)	27(93.1)		

## 4 Discussion

### 4.1 Interventions based on the BCW theory can improve patients’ health promotion behaviors

Our study demonstrated that the BCW-based intervention significantly improved health promotion behaviors in women with PCOS experiencing infertility, with the experimental group showing a median increase of 25 points in PCOS-HPLP total score versus 11 points in the control group. This finding is consistent with previous theory-driven interventions. Guo et al. (32) reported a 51-point increase in HPLP-II scores over 6 months using a TTM-based intervention, a larger effect than ours. This discrepancy likely reflects their longer intervention duration and more intensive follow-up (booster sessions every 2–3 days), whereas our intervention relied primarily on weekly support. Additionally, their inclusion of face-to-face dietary counseling and individualized exercise prescriptions may have enhanced adherence. Nevertheless, our 12-week intervention achieved a meaningful categorical shift from “moderate” to “high” HPB levels, suggesting that the BCW framework may accelerate early behavioral gains by simultaneously addressing multiple behavioral determinants rather than guiding patients sequentially through stages of change (21).

The improvements in dietary nutrition and exercise dimensions in our study align with the findings of Delavar et al. (33), whose health coaching intervention significantly increased physical activity and improved lifestyle scores. Notably, both studies found that behavioral improvements preceded physiological indicators, suggesting that psychological and behavioral gains may require longer follow-up to translate into clinical outcomes.

The superiority of our intervention over routine care can be attributed to the BCW framework’s comprehensive design. First, the intervention enhanced capability through both education (PCOS knowledge, disease mechanisms) and practical skills training (food exchange lists, calorie tracking, exercise technique, relaxation methods) (21)—enabling patients not just to know what to do but how to do it. Second, it addressed opportunity through environmental restructuring: WeChat-based support platforms, weekly healthcare team-patient exchanges, peer support groups, and caregiver involvement created a sustained supportive environment. Third, it strengthened motivation through education linking healthy behaviors to fertility outcomes, motivational interviewing techniques, caregiver encouragement, and peer modeling. This three-pronged approach explains why the experimental group outperformed the control group in dietary nutrition, exercise, and health responsibility—dimensions that require not only knowledge but also sustained behavioral effort.

The clinical relevance of these improvements is substantial. The shift from moderate to high HPB levels reflects tangible improvements in patients’ daily health practices—better dietary adherence, increased physical activity, and greater engagement with preventive health behaviors. These changes, if sustained, may contribute to improved metabolic parameters, menstrual regularity, and cardiovascular risk reduction, as recommended by international PCOS guidelines (11). Our findings underscore that practical skill-building (e.g., meal planning, exercise technique) is more effective than passive education alone, suggesting that clinical programs should allocate resources to structured behavioral training rather than relying solely on information provision.

### 4.2 Interventions based on the BCW theory can enhance patients’ self-efficacy

Our study found that the BCW-based intervention significantly improved self-efficacy in the experimental group (from 26.14 ± 5.05 to 29.79 ± 4.70, *p* < 0.001), while the control group showed no significant change. This finding corroborates the results of Young et al. (34), whose mindfulness-based program significantly improved nutrition and physical activity self-efficacy, and those of Goodarzi et al. (35), who reported significant self-efficacy improvements using the 5A’s model. The consistency of these findings across diverse theoretical frameworks suggests that structured, patient-centered behavioral support—regardless of specific theory—can effectively enhance patients’ confidence in managing their condition.

The mechanisms underlying our self-efficacy improvement are rooted in the BCW framework’s intervention functions (21). Self-efficacy, as a core component of reflective motivation in the COM-B model, was targeted through multiple pathways: education provided foundational knowledge; persuasion via motivational interviewing reinforced positive beliefs; incentivization through caregiver encouragement provided external reinforcement; and modeling via peer success stories offered vicarious experiences. Notably, the combination of these functions addressed the multiple sources of self-efficacy simultaneously, creating a synergistic effect that may explain why the experimental group’s improvement significantly exceeded that of the control group.

Our findings complement cross-sectional evidence from Guo et al. (36), who identified self-efficacy as a mediator between health literacy and quality of life in women with PCOS, and from Rzońca et al. (37), who found self-efficacy positively correlated with illness acceptance. While those studies described associations among these constructs, the present study provides interventional evidence that self-efficacy is a modifiable factor that can be actively enhanced through a structured, theory-driven program. This is consistent with the findings of Guo et al. (38), who identified self-efficacy as a significant predictor of health promotion behaviors in women with PCOS (*β* = 0.938, *p* = 0.002). Taken together, these observational and interventional findings collectively suggest that self-efficacy is both a relevant correlate of health outcomes and a viable target for behavioral intervention in this population, though formal mediational analyses in future studies would be needed to confirm the causal pathway from self-efficacy gains to behavioral change.

Clinically, the 3.97-point improvement in self-efficacy, though modest in absolute terms, is meaningful because self-efficacy is a robust predictor of long-term behavioral maintenance. Even small increments can differentiate between patients who sustain lifestyle changes and those who relapse. In chronic disease management, this distinction is critical, as PCOS requires lifelong adherence to healthy behaviors (11). Our findings suggest that clinicians should assess and actively cultivate patients’ self-efficacy as part of routine care, rather than assuming it will improve spontaneously through education alone.

### 4.3 The impact of intervention based on BCW theory on the pregnancy rate of patients

The experimental group showed a higher pregnancy rate (12.1% vs. 6.9%) during the 12-week intervention, but this difference was not statistically significant (*p* = 0.792). The lack of significant difference may reflect that most behavioral intervention studies in women with PCOS have not been designed or powered to detect pregnancy differences, as this outcome requires larger samples and longer follow-up than behavioral or psychological endpoints.

The absence of statistical significance can be explained by two factors. First, our sample size (66 participants) was calculated based on the primary outcome (HPB scores), not pregnancy rates. A post-hoc analysis suggests that detecting a 5.2% absolute difference with 80% power would require a substantially larger sample. Second, the 12-week intervention period may be insufficient to translate behavioral improvements into reproductive outcomes. Pregnancy represents the endpoint of a multi-step pathway, progressing from behavioral adherence to improved metabolic and endocrine function, then to restored ovulation, and eventually to successful conception. Each step requires time, and cumulative effects often demand longer observation periods than behavioral gains. Previous research by Oberg et al. (39) suggests that health promotion behavioral interventions in women with PCOS often require longer intervention and follow-up periods to demonstrate pregnancy benefits.

Despite the lack of statistical significance, the 5.2% absolute difference is clinically meaningful. In fertility medicine, an absolute increase of this magnitude would be considered meaningful—one additional pregnancy for approximately every 19 treated women. Therefore, the observed trend, while not statistically significant, justifies larger, adequately powered trials with extended follow-up.

### 4.4 Limitations

This study has several limitations. First, the sample size was calculated based on the primary outcome (HPB score) rather than the secondary outcome (pregnancy rate); therefore, the study was underpowered to detect differences in pregnancy outcomes, and the non-significant finding should be interpreted with caution. Second, the anticipated mean difference used in the sample size calculation was based on clinical experience rather than formal pilot data. Third, the primary outcome and one secondary outcome were assessed using self-report scales, which are subject to social desirability and recall biases. Fourth, due to the behavioral nature of the intervention, blinding of participants was not feasible, which may have introduced performance and expectancy biases; however, outcome assessors and data analysts remained blinded. Fifth, the 12-week intervention period may be insufficient to capture clinically meaningful improvements in pregnancy outcomes. Finally, this was a single-center study, which may limit the generalizability of the findings. Additionally, the internal consistency of the health responsibility and interpersonal support dimensions of the PCOS-HPLP scale was suboptimal in our sample; therefore, findings involving these two dimensions should be interpreted with caution. Future multi-center studies with larger samples, objective outcome measures, and extended follow-up are warranted to confirm our results and to further examine the scale’s measurement properties in this population.

## 5 Conclusion

This study suggests that the BCW-based HPB intervention program appears to be a promising approach for improving health promotion behaviors and self-efficacy in PCOS patients with infertility over a 12-week period. However, given the limited sample size, the short follow-up duration, and the non-significant difference in pregnancy rate, these findings should be interpreted as preliminary and require confirmation in larger, more robust trials. Nonetheless, the observed improvements in behavioral and psychological outcomes highlight the potential value of applying a systematic behavioral framework to support this population. Future multi-center studies with extended follow-up are needed to confirm the long-term sustainability of these benefits and to evaluate whether longer intervention durations can translate into clinically meaningful improvements in reproductive outcomes.

## Data Availability

The raw data supporting the conclusions of this article will be made available by the authors, without undue reservation.
